# Improving surgical skills with feedback: directly-observed versus video-recorded practice

**DOI:** 10.1186/s12909-023-04635-0

**Published:** 2023-09-11

**Authors:** Kasaya Tantiphlachiva, Cherdsak Iramaneerat, Tripop Lertbunnaphong

**Affiliations:** 1 Department of Surgery, Faculty of Medicine, Chulalongkorn University, 1873 Rama IV Road, Lumphini, Prathumwan, Bangkok, 10330 Thailand; 2 Department of Surgery, Faculty of Medicine Siriraj Hospital, Mahidol University, Bangkok, Thailand; 3 Department of Obstetrics and Gynecology, Faculty of Medicine Siriraj Hospital, Mahidol University, Bangkok, Thailand

**Keywords:** Surgical skill, Skill teaching, Feedback, Motivation, Video-feedback

## Abstract

**Objective:**

This study aimed to compare two methods of feedback: verbal face-to-face feedback after direct observation (*F2F-feedback*) *versus* electronic-written feedback after observation of recorded-VDO of student’s performance (*VDO-feedback*), in terms of effectiveness in improving skill, effects on motivation and satisfaction.

**Background:**

Medical schools are responsible for teaching and ensuring proficiency of basic surgical skills. Feedback is effective in developing psychomotor skills; by providing information of learner’s current performance, how to improve, and enhancing motivation.

**Materials and method:**

Fifty-eight medical students (3^rd^– 4^th^ year) were trained to perform vertical mattress suture in small groups. Then, during 6-week period of self-directed practice, students were randomized into group1 *VDO-feedback* (male:female = 21:8) and group 2 *F2F-feedback* (male:female = 20:9). Feedbacks were provided once every 2 weeks (Week2, Week4). End-of-rotation OSCE was at Week6, and retention tested was at Week8. Performance checklist (Cronbach’s Alpha 0.72) was used to assess skill at 4 timepoints; pre- and post- small group learning, OSCE, and retention phase. Questionnaire was used to assess motivation, learning strategies and satisfaction (Cronbach’s Alpha 0.83).

**Result:**

After in-class learning, further significant improvement of skills could be gained by both F2F- and VDO- feedbacks (*p* < 0.0001). Both could similarly retain skill for at least 4 weeks later without additional practice. Self-efficacy, test anxiety, and cognitive strategies scores were significantly increased in both groups (*p* < 0.05). Extrinsic motivation was increased in VDO-feedback group. No difference in satisfaction between groups was observed.

**Discussion and conclusion:**

VDO-feedback could be alternative to F2F-feedbacks for basic surgical skill training when limitation for simultaneous meeting of teacher and students occurs.

**Trial registration:**

This study has been registered to Thai Clinical Trial Registry (WHO International Clinical Trial Registry Platform) on 11/07/2023 (TCTR20230711005).

**Supplementary Information:**

The online version contains supplementary material available at 10.1186/s12909-023-04635-0.

## Introduction

Basic surgical skills are essential for physicians in their clinical practice. Medical schools are responsible for teaching and ensuring proficiency of these skills in their undergraduates while students are responsible for self-directed practice [1]. In our institute, a 2-h small-group teaching session, containing skill demonstration and practice under supervision in part-task models, has been used. Students may gain additional experience in real patients in the clinic, operating room, and emergency room. Further self-directed practice is encouraged by providing a take-home “suture practicing kit”. The skills would be assessed as a part of the end-of-rotation summative Objective Structured Clinical Examination (OSCE). Scores of 65–75% were seen in the past few years, which were unsatisfactory. Explanation may be inadequate learning or inadequate practice. Firstly, we failed to reach the optimal teacher to student ratio for motor skill training of 1:4 [2]. This is a limitation in most medical schools. Secondly, inadequate further self-directed practice was likely due to a lack of students’ external motivation. Students might internally be motivated to be a good and skillful doctor but the drive to repeatedly practice the skills might not be steadily high enough.

The critical step that could be added during this self-directed psychomotor skill practice period is “feedback” [3–6]. “Feedback” is a teaching strategy which specific information about learner’s performance, as compared to the established standard, is given to the learner with aims to increase learner’s motivation and improve learner’s performance [3]. For procedural skills, feedback is one of Gagne’s critical learning conditions [4] and a part of Ericsson’s deliberate practice condition to acquire expert performance [5]. Classically, feedback is given verbally right after direct observation of skills. Other ways which feedback can be conveyed are via paper, video, audio and digital platform [7]. Among these, video-recorded practice and feedback (VDO-feedback) is attractive because (1) asynchronous assessment and feedback was possible [7, 8] when there is a limited number of experts available. (2) individualized feedback making teacher to student ratio 1:1. (3) self-review is possible (4) Performing task without presence of assessor would reduce stress and anxiety [9] and (5) Ease of production of self-recording VDO using smartphone or tablet [9]. Another role of feedback is to improve students’ *motivation* in learning [10, 11]. According to self-determination theory, to enhance intrinsic motivation, 3 innate psychological needs, namely competence, autonomy and relatedness, should be satisfied [12]. Students may be intrinsically motivated by a need to achieve physician’s surgical skill competency which extrinsically related them to be a part of medical society. Self-directed practice may provide a feeling of autonomy. Feedback by a surgical teacher about their skill would support their competence and feeling of relatedness. To assess learning, which is a relatively permanent change in a person’s capability to perform a skill [13], 2 measures could be used; (1) comparison of the “entering behavior” to the “post-training behavior” [4], and (2) assessment of “skill retention” which reflects changes in behavior over time [14, 15].

Whether VDO-feedback is as effective as standard face-to-face(F2F) verbal feedback, in terms of improvement of surgical skill is not definitely conclusive. This study aimed to compare two types of feedback in improving surgical skill. Hypotheses of this study were (1) there are differences in performance score improvements after surgical skill practice with F2F- and with VDO- feedback (2) There are differences in retention scores between students who had surgical skill practice with F2F- and with VDO-feedback (3) there are differences in changes of motivation and learning strategies scores between students received different feedbacks, and (4) there are differences in satisfaction scores after surgical skill practice with F2F-and VDO-feedback.

## Materials and methods

### Subjects

The 4^th^-year medical students, just before the start of surgical rotation; and the 3^rd−^-year students, just before the start of clinical years, were voluntarily enrolled via advertisement. The period of study was from 28th December 2020 to 9th April 2021. Students who were interested were given a simple, clear, and thorough explanation about the purposes of the study, roles and benefits of participants, and additional inquiry was allowed until satisfied. They were free to deny, participate, or drop out of the study at any time. Informed consents were obtained prior to participation.

Half of the 4^th^ and half of the 3^rd^ year students were randomly assigned into Group 1: face-to-face feedback and Group 2: video feedback.

### Flow of the study

The study was divided into 2 phases: skill learning phase and intervention phase.

#### Skill learning phase

During the period of 4 or 6 weeks (for 3^rd^ and 4^th^ year, respectively), students would participate in 2–3 sessions of small group teaching of “vertical mattress” suturing. Simulated skin pad with a 3-cm cut wound and 4/0 Polyamide threads were used.

#### Intervention phase

##### Face-to-face feedback group (F2F-feedback)

Onsite sessions with a surgical teacher (a researcher) were scheduled every other week for 2 times. During F2F-feedback session, students were asked to perform wound closure using vertical mattress technique. A teacher gave verbal feedback after a few stitches had finished. Each feedback lasted 20 min.

##### Video-feedback group (VDO-feedback)

Students were taught how to VDO-record their performance using their smartphone and the provided smartphone holder. They were asked to submit a VDO clip demonstrating wound closure with 3 stitches of vertical mattress technique, every other week for 2 times. The submission was done by uploading the VDO-clips to Google Drive™ and sharing them with a surgical teacher (a researcher) via university email. After reviewed, the feedback message was typed and sent back via their email within 1–2 days.

During the 6 weeks of intervention phase, students were asked not to share or discuss about skill training and feedback with their friends. They were not allowed to assess other sources of skill training including additional classroom training, skill workshop, accessing online resources other than the provided demonstration VDO.

### Performance assessment

**Fig. 1:**
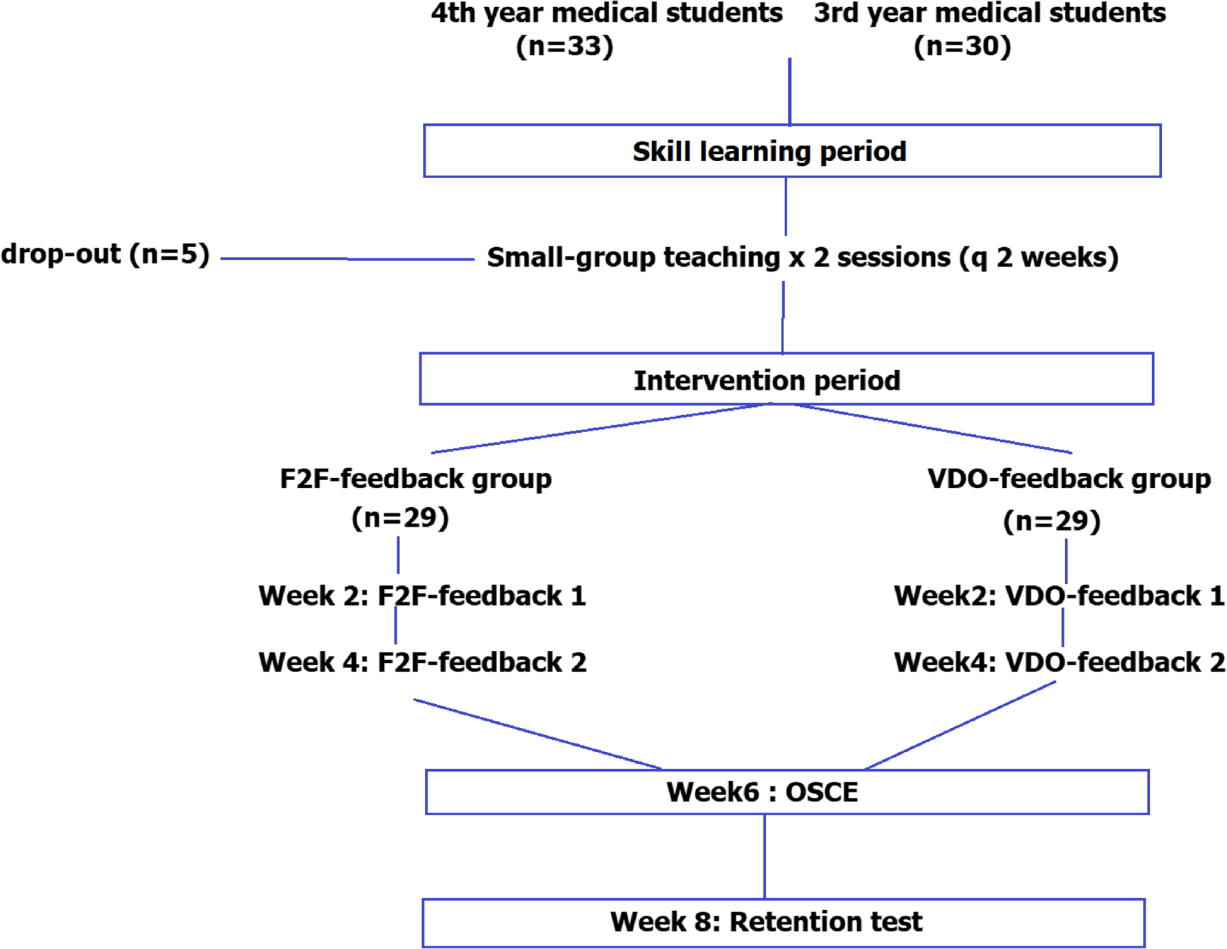
Flow of the study

For assessment purposes, students were asked to perform 2 vertical mattress stitches to close a standard 3-cm cut wound on a simulated skin pad within 4 min. Assessment of suturing skill performance were carried out at 4 timepoints; pre-learning test, post-learning test, OSCE, and retention test. For the 4^th^ year students, this item was one of 12 OSCE stations at the end-of-rotation. The retention test was performed 2 weeks after the end-of-rotation when there was no additional practice or teaching (Fig. 1). This point of time was selected because it was 4 weeks from the last feedback and before the students would further gained additional clinical experience.

Each performance was VDO-recorded and later evaluated using a developed performance checklist which is developed from the Objective Structured Assessment of Technical Skills (OSATS) [16]. Items to assess instrument handling, respect of tissue, suture techniques and tissue approximation were rated as perfect, partially correct, or incorrect with appropriate weighted scores. A total sum of 100 points indicated perfect expert skill. The questionnaire was validated for its content by 3 experts and was tried in the 6^th^ year medical students (*n* = 61). They were asked to perform the same skill which would be VDO-recorded. Two independent surgical teachers were asked to rate their skills using the questionnaire. Internal consistency reliability (Cronbach’s alpha) was 0.66 and inter-rater reliability was 0.55 (95%CI 0.22–0.74, *p* = 0.002). The inter-rater reliability for the part of instrument handling was 0.62 (95%CI 0.35–9.78, *p* < 0.001) and for the part of vertical mattress technique was 0.66 (95%CI 0.41–0.81, *p* < 0.001). For the part of knot tying, the inter-rater reliability was only 0.20 (95%CI 0.39–0.53, *p* = 0.22).

### Assessment of motivation and learning strategies

A questionnaire to assess students’ motivation was derived from publicly available Motivated Strategies for Learning Questionnaire (MSLQ) by Pintrich et al. [17]. For motivation, 3 areas were measured: (1) *value* (intrinsic and extrinsic orientation, task value), (2) *expectancy* (learning control belief, self-efficacy) and (3) *affect* (test anxiety). Learning strategies were also evaluated which divided into 3 domains: (1) *cognitive* (a. rehearsal, b. elaboration, c. organization, and d. critical thinking), (2) *metacognitive* (a. planning b. monitoring and c. regulating) and (3) *resource management* (a. managing time and study environment, b. effort management, c. peer learning and d. help-seeking). Thirty-one items were selected to measure across these topics, using a 7-point Likert scale; ranging from 1 = totally disagree to 7 = totally agree. Internal consistency reliability was 0.84. Questions were modified to match the context of undergraduate medical students. Students were asked to fill this questionnaire twice, at the start-of-course (pre-learning) and at the end-of-course (post-OSCE). Satisfaction to feedback intervention (6 mini-items, 7-point Likert scale), demographic data and practice log were also recorded. Range of scores depended on the number of items in the questionnaires, i.e., minimum was the number of items and maximum was the number of items multiply by 7.

### Ethical consideration

Data obtained from participants, both by questionnaires and performance tests was kept confidential. Their identity would not be disclosed, except for the purpose of summative assessment at the end-of-surgical rotation of the 4th year medical students. By design, students in both groups benefited from the feedback intervention. This study has been approved by Institutional Review Boards of Human Research Protection Unit, Faculty of Medicine Siriraj Hospital, Mahidol University (no. 887/2563(IRB2)) and the Ethical Review Board, Faculty of Medicine, Chulalongkorn University (no.764/63). This study has been registered to Thai Clinical Trial Registry (WHO International Clinical Trial Registry Platform) on 11/07/2023 (TCTR20230711005).

### Statistical analysis

SPSS for Microsoft Windows version 22. Statistical significance was set at *p*-value of equal or less than 0.05. Descriptive statistics were used for demographic data. Comparison of performance scores between groups at each timepoint were done by independent sample t-test. Comparison in each group between 2 different timepoints were done by pair sample t-test. Difference in performance scores among post-learning, OSCE and retention timepoints were done by repeated measure ANOVA.

## Results

Of the initial 63 interested students, 5 withdrew after small-group learning, leaving 58 eligible students (4^th^: 3^rd^ year 28:30) to complete the study. Half of each year were randomly assigned into Group 1: face-to-face-feedback (4^th^: 3^rd^ year 14:15) and Group 2: video feedback (4^th^: 3^rd^ year 14:15). There was no difference between students’ gender, mean age, grade point average (GPA), handedness, and previous skill training experience between groups (Table 1).

**Table 1:** Demographic data of F2F- and VDO-feedback groups

	**VDO-feedback** (*n* = 29)	**F2F-feedback** (*n* = 29)	Total (*n* = 58)	*p*-values
Male: Female	21:8	20:9	41:17	0.773
Age (years, mean ± SD)	21.1 ± 1.5	21.0 ± 0.8	21.0 ± 1.2	0.471^##^
GPA	3.35 ± 0.43	3.47 ± 0.39	3.41 ± 0.41	0.232^##^
Right: Left-handed	28:1	26:3	54:4	0.611^#^
Year4: Year3	14:15	14:15	28:30	1.000^##^
Previous skill training	4^a^	0	4	0.112^#^

^#^Fisher’s exact test

^##^Mann–Whitney U test

^a^All were the 3^rd^ year students

Performance scores were not different between groups at 3 timepoints; pre-learning, OSCE and retention. However, at post-learning point, F2F-feedback group had significantly higher scores than the VDO-feedback group. This could be related to the higher initial (pre-learning) score of the F2F-feedback group (Table 2).

**Table 2:** Comparison of mean performance test scores between groups at 4 timepoints (mean ± SD)

**Test**	**VDO-feedback**	**F2F-feedback**	***p*-values**
Pre-learning	44.1 ± 15.5	49.4 ± 14.7	0.186
Post-learning	81.3 ± 11.9	87.8 ± 8.7	0.022*
OSCE	93.2 ± 10.7	95.9 ± 4.6	0.222
Retention	93.6 ± 7.6	96.1 ± 5.3	0.145

^*^significant level at *p* < 0.05

**Fig. 2:**
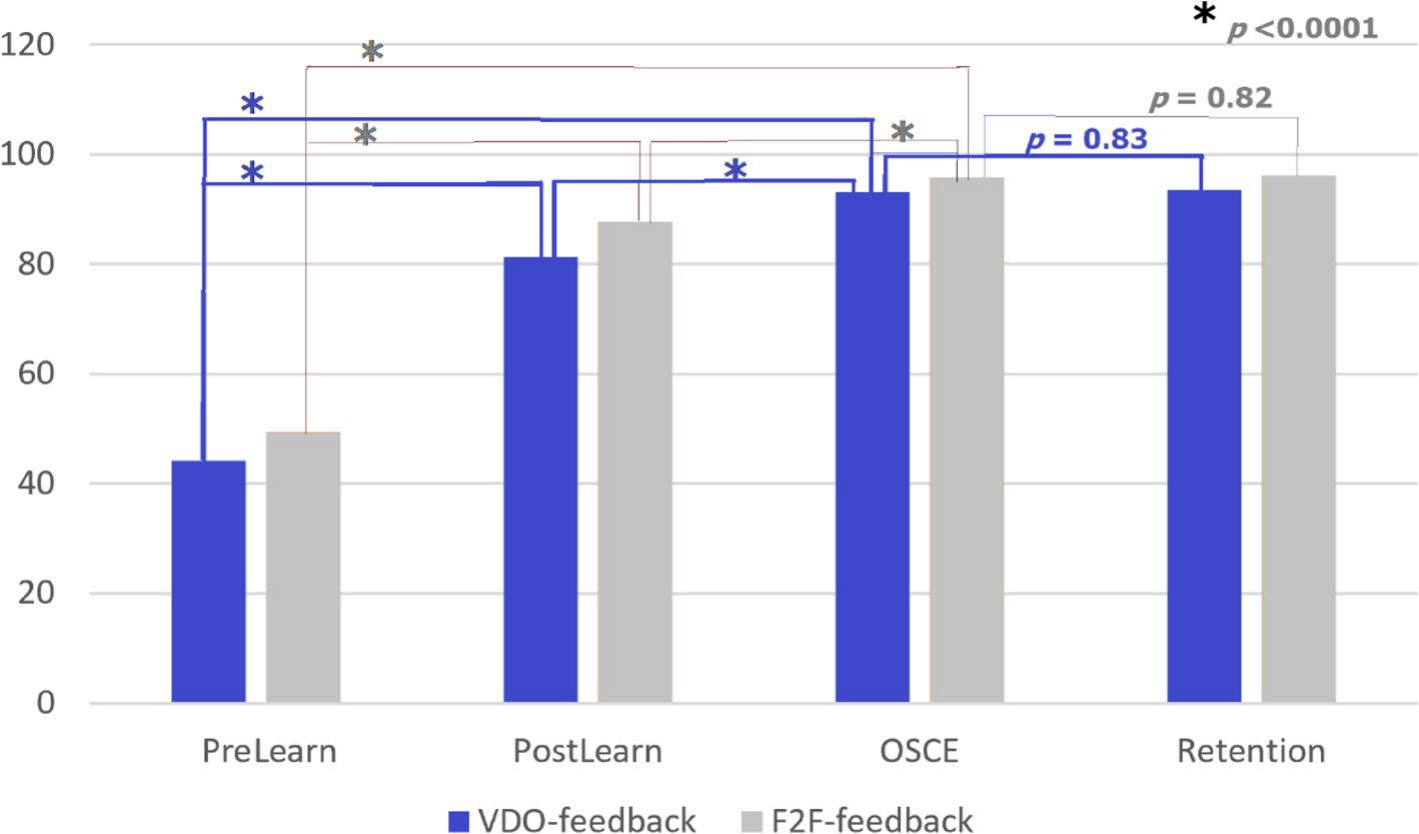
Barchart of mean performance test scores at 4 timepoints; prelearning, postlearning, OSCE and retention test

**Fig. 3:**
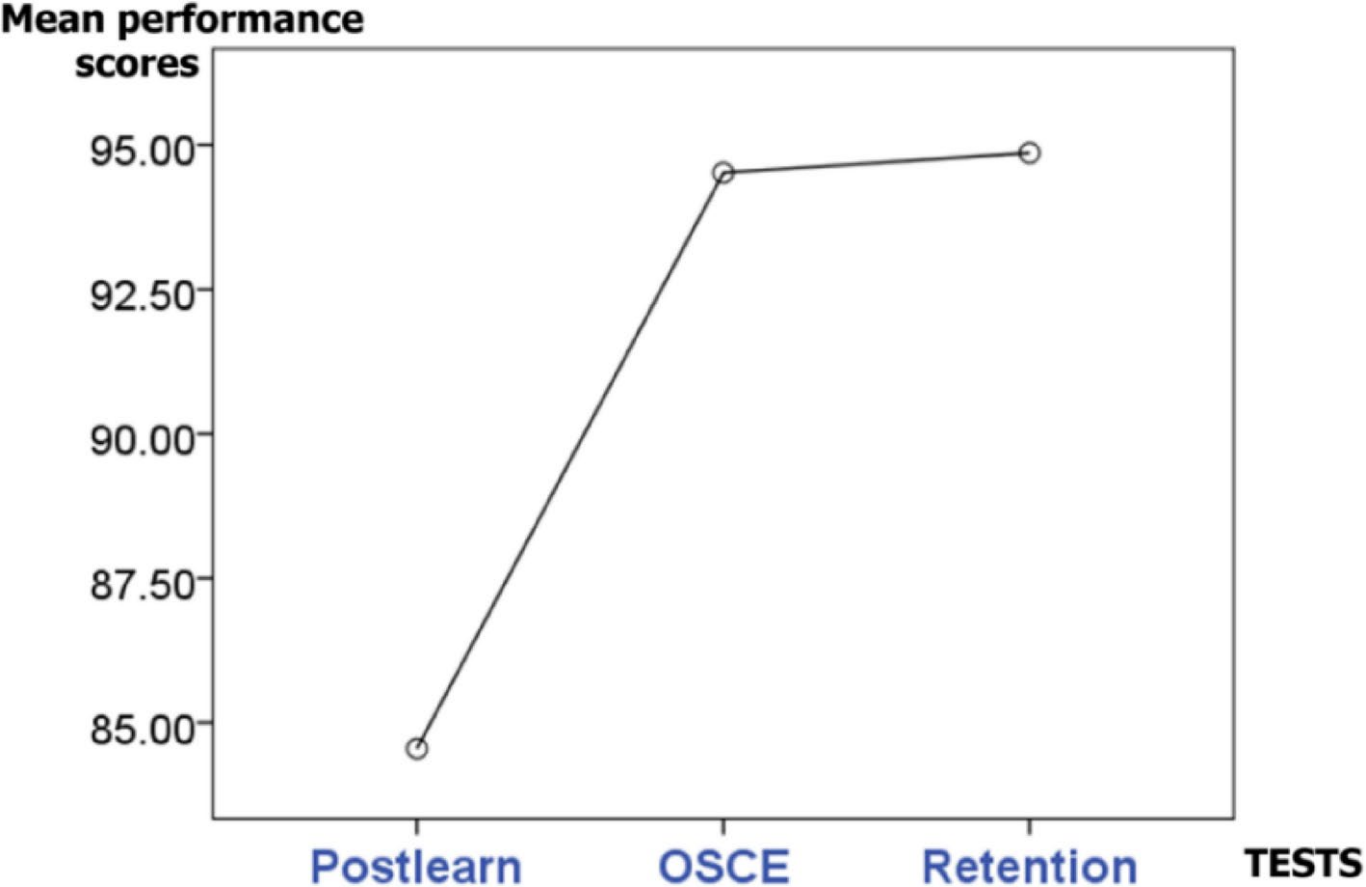
Mean performance test scores at postlearning, OSCE and retention timepoints

The higher mean performance scores could infer to improvement of surgical skills closer to the expert level. When scores between each pair of different timepoints were compared (Figs. 2 and 3), significant increase of score were seen after small group learning (pre-learning *vs*. post-post-learning), after feedback (post-learning *vs*. OSCE), and after small group learning + feedback (pre-learning *vs*. OSCE) with *p* < 0.05. These were found in both groups of feedback (Figs. 2 and 3). There was no difference between scores at retention and OSCE timepoints of both groups. Statistical power of the analysis was assessed by G*power 3.1.9.7 program. Effect size of 1.58 and power of 0.99 were seen. Repeated measure ANOVA with a sphericity assumed shows significant difference in the performance test scores among the post-learning point, OSCE, and retention test: F (2,144) = 42.63, *p* < 0.001. Significant difference were seen between post-learning and OSCE point (*p* < 0.001).

Motivation and learning strategies scores between pre-learning and post-OSCE timepoint were compared. Total scores and sum of scores of both motivation part and learning strategies parts were significantly increased in both groups (Table 3). There was no difference between groups in these scores. In the part of motivation, self-efficacy and test-anxiety scores were improved in both groups. Intrinsic motivation was unchanged in both group and extrinsic motivation was increased only in the VDO-feedback group. In the part of learning strategies, significant increase of cognitive and metacognitive strategies scores was improved in both groups. However, there were no different change in metacognitive self-regulation, management of time and study environment, and help-seeking strategies.

**Table 3:** Mean modified motivation and learning strategies questionnaire scores

**Components (possible scores)**	**VDO-feedback**	**F2F-feedback**	***p*-values**	**Total**
**Self-efficacy (4–28)**				
Pre-learning score	15.9 ± 4.1	17.3 ± 4.0	0.183	16.6 ± 4.2
Post-OSCE score	18.3 ± 4.1	19.4 ± 3.4	0.298	18.9 ± 3.8
	0.001*	< 0.0001*		< 0.001*
**Intrinsic motivation (7–49)**				
Pre-learning score	33.2 ± 5.2	35.2 ± 4.6	0.130	34.0 ± 5.0
Post-OSCE score	33.7 ± 4.7	34.9 ± 5.1	0.370	34.2 ± 4.9
	0.486	0.802		0.730
**Extrinsic motivation (4–28)**				
Pre-learning score	14.1 ± 4.2	14.0 ± 3.0	0.943	14.0 ± 3.7
Post-OSCE score	15.2 ± 5.0	14.8 ± 2.9	0.720	15.0 ± 4.0
	0.020*	0.508		0.018*
**Test anxiety (2–14)**				
Pre-learning score	8.0 ± 2.8	7.5 ± 2.2	0.427	7.8 ± 2.5
Post-OSCE score	9.2 ± 3.4	9.0 ± 2.3	0.820	9.1 ± 2.9
	0.016*	0.0007*		0.001*
**Cognitive and metacognitive strategies: Rehearsal, elaboration, organization (2–14)**				
Pre-learning score	8.4 ± 1.9	9.5 ± 1.9	0.04*	9.0 ± 2.0
Post-OSCE score	9.8 ± 1.8	10.0 ± 1.6	0.66	9.9 ± 1.7
	< 0.0001*	0.029*		< 0.0001*
**Metacognitive self-regulation (3–21)**				
Pre-learning score	13.1 ± 2.2	15.1 ± 2.7	0.003*	14.1 ± 2.6
Post-OSCE score	13.7 ± 2.8	14.4 ± 2.9	0.365	14.1 ± 2.9
	1.00	0.924		1.00
**Management of time and study environment (5–35)**				
Pre-learning score	24.3 ± 3.7	24.4 ± 3.3	0.912	24.3 ± 3.5
Post-OSCE score	25.2 ± 3.4	24.1 ± 2.7	0.207	24.7 ± 3.1
	0.459	0.866		0.459
**Help-seeking strategies (6–42)**				
Pre-learning score	29.6 ± 5.0	30.8 ± 4.0	0.367	30.2 ± 4.6
Post-OSCE score	31.7 ± 5.0	31.0 ± 4.6	0.611	31.4 ± 4.8
	0.288	0.124		0.288
**Total score (32–224)**				
Pre-learning score	146.2 ± 16.7	153.5 ± 21.4	0.090	149.8 ± 16.2
Post-OSCE score	191.6 ± 21.4	192.8 ± 16.7	0.807	192.2 ± 19.1
	< 0.0001*	< 0.0001*		< 0.001*
Mean difference of scores^a^	45.3 ± 12.7	39.3 ± 13.6	0.090	
**Sum of Motivation Scores (15–105)**				
Pre-learning score	71.3 ± 10.2	74.2 ± 8.2	0.245	72.7 ± 9.5
Post-OSCE score	76.6 ± 10.6	78.0 ± 8.4	0.585	77.3 ± 9.5
	0.007*	< 0.0001*		< 0.001*
Mean difference of scores^a^	4.1 ± 7.3	2.6 ± 5.4	0.393	
**Sum of Learning Strategies Scores (16–112)**				
Pre-learning score	75.2 ± 9.4	79.7 ± 9.7	0.084	77.5 ± 9.8
Post-OSCE score	80.3 ± 9,9	78.9 ± 8.1	0.540	79.6 ± 8.3
	0.151	0.082		0.027*
Mean difference of scores^a^	5.1 ± 8.3	0.4 ± 9.5	0.059	

^*^Statistical significance at *p* < 0.05

^a^Difference scores = Post-learning score – Pre-learning score

At the end of rotation (post-OSCE), satisfaction was assessed in 6 topics (Table 4). Students who participated in VDO-feedback group had slightly higher satisfaction scores towards the intervention, in terms of promotion of comprehension of instrument handling and suturing technique, time-to-feedback, convenience of access to instructor and clarity of perceived information. However, they were less likely to recommend this type of feedback to others.

**Table 4:** End-of-course students’ satisfaction scores

**Satisfaction components**	**VDO-feedback**	**F2F-feedback**	***p*-values**
The course promoted comprehension of surgical instrument handling	6.1 ± 0.8	6.0 ± 0.9	0.757
The course promoted comprehension of suturing technique	6.1 ± 0.8	6.0 ± 0.8	0.619
Time-to-feedback is appropriate	6.0 ± 0.9	5.1 ± 0.9	0.554
Access to instructor is convenient	6.1 ± 1.1	5.8 ± 1.0	0.228
Perceived information is clear	6.0 ± 1.1	5.9 ± 1.0	0.800
Likelihood of recommendation to others	5.4 ± 1.3	5.9 ± 0.9	0.128
**Total satisfactory scores**	36.1 ± 4.7	35.4 ± 4.6	0.597

## Discussion

This study addresses the problem of lower-than-expected student’s performance of surgical skill. The proposed explanations were inadequate periodic practice and/or inadequate learning. Compared to the previous small-group teaching with informal feedback, this study had added formal feedback intervention, by either face-to-face or video- styles. Students were scheduled for 2 regular feedbacks during the period that they were supposed to carry out self-directed practice of the procedural skill. Feedback interventions during this period had been proven to improve students’ performance significantly after in-class small group learning. There was no significant difference between F2F- and VDO-feedback in this improvement. Both interventions could retain skill performance up to at least 4 weeks later without further practice. Feedback had been demonstrated to be moderately effective in simulation-based procedural skill teaching [18]. For successful outcome in behavior changing, an actionable feedback model was proposed by Hysong et al. [19]. The characteristics of effective feedback were timeliness, personalization, non-punishing, and customizability. Both feedbacks seem to have these characteristics. In terms of timeliness, F2F-feedback was immediate, but VDO-feedback was later but withing 1–2 days. Thus, this duration is likely acceptable.

Both types of feedback were shown to improve motivation and learning strategies scores. A student who is motivated would use more effective learning strategies to achieve his/her academic goal [10]. Intrinsic goal orientation (or intrinsic motivation) was found to have significant effect on learning strategies [20, 21]. Both intrinsic motivation and learning strategies directly affects learning outcome [21]. Extrinsic motivation had less effect on student’s performance than intrinsic motivation [20]. Self-efficacy in learning as shown by self-confidence level had been improved significantly after both types of feedback. Intrinsic motivation score was not changed by the feedbacks, but the extrinsic motivation was significantly increased with VDO-feedback. This may be explained by submitting VDO-record of their performance, they would get an “email” back. In the email, some compliments were added together with the instruction of how to improve which they could review many times. In the F2F-feedback, the verbal compliment and instruction might not be as “objective” as the written ones. Increased test anxiety was supposed to impair student’s performance [22]. However, there were evidences showing benefits of stress to academic [23], and physical performance [24]. Optimal amount of anxiety would drive students to practice for better performance. Although, some evidence showed that anxiety did not seem to affect students’ performance during simulation skills-based learning [25], or OSCE [26]. The absolute amount of optimal anxiety was not known. After the intervention, students had more test anxiety score which was beneficial for the students.

Cognitive and metacognitive strategies inferred to how a student memorized and elaborated information he/she learnt. These comprised rehearsal; a simple recital of procedure steps, elaboration; processes to incorporate the new skills to previously known skills, and organization; a learner’s attempt to construct pattern(s) of new knowledges. At baseline, F2F-feedback group showed significantly higher cognitive and metacognitive strategies scores. However, this difference had been eliminated after feedback intervention, and the post-intervention scores had significantly improved from pre-intervention. The same results were seen in metacognitive self-regulation scores, which is how learners manage themselves in practicing and performing skills. Improvement in these scores were found after both types of feedback and seemed to correlate with the improved performance scores.

Students who participated in VDO-feedback group had slightly higher satisfaction scores towards the intervention, in terms of promotion of comprehension of instrument handling and suturing technique, time-to-feedback, convenience of access to instructor and clarity of perceived information. However, they were less likely to recommend this type of feedback to others. Further study is needed to confirm and explore this finding. Our hypothesis was that VDO-recording is an additional workload for students. Cook et al. [27] had reported reliable association between MSLQ scores with knowledge score and satisfaction scores. This confirms our findings. With the similar efficacy of both feedback intervention on improving basic surgical skill, motivation, learning strategies and satisfaction, VDO-feedback could be an alternative to the classic F2F-feedback in the situation that there is a limited time and space for simultaneous presentation of teacher and students. For example, 1) Clinician-as-a-teacher with limited teaching schedule 2) Clinical-year-apprentice with tight schedule 3) Institute with limited official teaching space and 4) When social distancing is warranted.

Some possible drawbacks of VDO-feedback for procedural skill teaching are (1) learners cannot communicate with teachers in the real time and (2) teachers need to be good at writing communication [9]. Also, things to consider before applying the technique for procedural skill teaching are; 1) A part-task simulation which easy-to-find and easy-to-set up tools may be more suitable. 2) Before students can practice on their own, didactic and/or formal training may be required to ensure correct steps. 3) Students would be better motivated if the feedback is “on-time” and “in-time”. That is a regular interval is scheduled for VDO-submission and a teacher should review and feedback within 24–48 h. 4) Students and teachers should agree on the mode of training.

## Conclusion

This study has compared effectiveness of asynchronous electronic feedback after VDO-observation and synchronous face-to-face feedback after direct observation of student’s performance of basic procedural skills. Both feedback interventions were effective in improving and retaining student’s performance skill and also improved student’s motivation and learning strategies. Students shows high satisfaction scores for both interventions. VDO-feedback could be an alternative to face-to-face feedback in teaching procedural skills in the situation when simultaneous presentation of teacher and students are limited, by time, space, and social situation. Further study of VDO-feedback in other context; more complex procedural skill, in other specific group of students, and with different feedback protocol are encouraged.

### Supplementary Information

**Additional file 1.**

## Data Availability

The datasets generated and/or analysed during the current study are not publicly available due to these data also included the students’ examination scores which belong to the Faculty of Medicine, Chulalongkorn University. The retrospective retrieval of data needs permission process to the dean of the Faculty of Medicine, Chulalongkorn University, and may be available from the corresponding author on reasonable request.
