# Supplementary material for: Improving surgical skills with feedback: directly-observed versus video-recorded practice
Source: BMC Med Educ. 2023 Sep 11;23:660. doi: 10.1186/s12909-023-04635-0 (PMC10496316; doi:10.1186/s12909-023-04635-0)
Supplement: Supplementary file 1 — Additional file 1. [file 12909_2023_4635_MOESM1_ESM.docx]

**APPENDIX A**

**PERFORMANCE CHECKLIST**

**Rating scale for vertical mattress x 2 stitches (4 minutes)**

**Name ....................................................................... No. .........................**

| **Steps** | **Manners** | **Performance** | | **Not done/ incorrect** |
| --- | --- | --- | --- | --- |
|  |  | **Correct** | **Not perfect** |  |
| **1. Needle handling** | - Needle was held at the tip of the needle holder (2 points = not at the tip but practical)- Needle was held at 2/3 from its tip (2 points =1/2 from its tip)- Needle was held perpendicularly to the needle holder - Needle holder was held by inserting the thumb **and** the ring finger into its rings  - Needle holder was stabilized by the index finger **and** was locked | 4 4  2  4  4 | 2  2  x  2  2 | 0  0  0  0  0 |
| **2. Forceps handling** | - Tooth forceps was correctly held in the other hand(pen-handling using thumb and index finger or thumb and index + middle fingers) | 4 |  | 0 |
| **3. Vertical mattress suturing** | - Passing the needle 6-10 mm away from the wound edges, on either side of the wound- A needle tip was always placed perpendicularly to the skin (2 points=sometimes) - Move the needle along its curve | 8  4  2 | 4  2  X | 0  0  0 |
|  | -The depth of suture covered the whole depth of the wound by bringing the needle out in the middle of the wound first and then went back from the bottom to the opposite side of the wound, using forceps and needle holder to manage the needle (4 points=**cover** the bottom of the wound in one bite, 0 point=could not cover the bottom of the wound) | 8 | 4 | 0 |
|  | Re-handling the needle in the backhand position without using bare hand | 4 |  | 0 |
| Backward | - Holding needle holder in backhand position- Placing stiches 1-2 mm away from the wound edges on either side | 4 4 | X  2 | 0  0 |
|  | - The wound edge was not cut-through by the suture material | 4 |  | 0 |
| **4. Knot-tying with instruments** | - Using the needle holder to make the surgical knot(4 points = square knot, 0 point= granny knot/other/not tie) | 8 | 4 | 0 |
|  | - Made 3 throws | 4 | 2  (2 throws) | 0  (1 or >4 throws) |
|  | The wound edges were brought closer and approximated properlyNot too tightened | 8 4 | 4  X | 0  0 |
|  | The stitches was perpendicular to the wound edge---- I ----- The suture was cut at 0.5-1.0 cm above the knot | 4 4 |  | 0  0 |
|  | Minimal tissue injury | 4 |  | 0 |
|  | Complete 2 stiches in time | 4 |  | 0 |
| Sum (Total 100 points) | |  |  | |
| Global rating scale (circle around the number)**1 2 3 4 5Novice Competent Expert | | Signature | | |
| 🞎 Fail 🞎 Borderline 🞎 Pass 🞎 Good Pass | |  |  |  |

**Novice means many unnecessary moves. Frequently stops, Unsure to next step. No anticipation of suture needs. Many sutures inaccurately placed.

Competent means Few unnecessary moves, Efficient time and motion. Demonstrates forward thinking and planning. Most sutures accurately placed.

Expert means Clear economy of motion. Obviously planned next steps and demonstrate fluidity in actions. Accurate suture placement.

**APPENDIX B**

The Modified Motivated Strategies for Learning Questionnaire (MSLQ).

**Please rate your opinion towards the following topics**

**(1 = Totally disagree และ 7 = Totally agree)**

|  |  | Totally disagree |  |  |  |  |  | Totally agree |
| --- | --- | --- | --- | --- | --- | --- | --- | --- |
|  |  | 1 | 2 | 3 | 4 | 5 | 6 | 7 |
| 1 | I am very good at procedural skill |  |  |  |  |  |  |  |
| 2 | I am very confident in performing procedural skills in manikins |  |  |  |  |  |  |  |
| 3 | I am very confident in performing procedural skill in the real patient |  |  |  |  |  |  |  |
| 4 | I have been interested in performing procedural skill long before becoming the 4^th^ year student |  |  |  |  |  |  |  |
| 5 | I think this method of learning procedural skill is interesting |  |  |  |  |  |  |  |
| 6 | Participating in this activity would help me learn procedural skill better than the regular teaching method |  |  |  |  |  |  |  |
| 7 | When I heard about this project, I felt very interested in this kind of procedural skill training |  |  |  |  |  |  |  |
| 8.1 | I believe that surgical procedural skill is very important for patient management |  |  |  |  |  |  |  |
| 8.2 | Procedural skill training will help me give better care to patients |  |  |  |  |  |  |  |
| 9 | Making good grades is the most important thing for me now |  |  |  |  |  |  |  |
| 10 | I will practice procedural skill in order to improve my total scores |  |  |  |  |  |  |  |
| 11 | My family is a great pressure that drive me to make good grades |  |  |  |  |  |  |  |
| 12 | It does not matter if I could not pass the procedural skill examination, as long as the summary score is “pass” |  |  |  |  |  |  |  |
| 13 | OSCE station that tests procedural skill makes me very nervous |  |  |  |  |  |  |  |
| 14 | I have palpitation, shaking hand and sweating during the procedural skill examination |  |  |  |  |  |  |  |
| 15 | I can picturize each step of the procedural skills clearly |  |  |  |  |  |  |  |
| 16 | I try to link the new information leant to my previous knowledge |  |  |  |  |  |  |  |
| 17 | In preparation for examination, I divide times for practicing procedural skills and reading didactic knowledge equally |  |  |  |  |  |  |  |
| 18 | I always spare time for practicing new procedural skill without anyone to tell me so |  |  |  |  |  |  |  |
| 19 | I believe that practicing procedural skills in manikin will make me better when practice in real patient. |  |  |  |  |  |  |  |
| 20 | Practicing procedural skills in manikins is not necessary if there is adequate number of real patients |  |  |  |  |  |  |  |
| 21 | Having a take-home procedural skill practicing kit gives me more sufficient time to practice, |  |  |  |  |  |  |  |
| 22 | Having a take-home kit make it convenient for me to review the steps of the procedure frequently |  |  |  |  |  |  |  |
| 23 | Having a take-home procedural skill practice kit obviates the necessity of leaning in the classroom |  |  |  |  |  |  |  |
| 24 | I can find a peaceful corner to practice procedural skill |  |  |  |  |  |  |  |
| 25 | If I’m not sure about the steps of procedural skills, I know where to find the answer |  |  |  |  |  |  |  |
| 26 | Having scheduled to meet the teacher helps me plan the learning of procedural skill better |  |  |  |  |  |  |  |
| 27 | Having scheduled to meet the teacher makes me practice procedural skills more often |  |  |  |  |  |  |  |
| 28 | Having scheduled to meet the teacher gives me opportunity to ask questions |  |  |  |  |  |  |  |
| 29 | I learn procedural skill better from my friends than from VDO or teacher |  |  |  |  |  |  |  |
| 30 | Having scheduled to meet the teacher is an unnecessary extra work |  |  |  |  |  |  |  |
| 31 | I can teach procedural skill to my friend |  |  |  |  |  |  |  |

Suggestion ....................................................................................................................................................................................................................................................................................................

..................................................................................................................................................

**Remark** These questions were modified from Pintrich PR, Smith DAF, Garcia T, McKeachie WJ. A manual for the use of the Motivated Strategies for Learning Questionnaire (MSLQ).

Available from: <https://files.eric.ed.gov/fulltext/ED338122.pdf> [Internet].
